# Comparative proteomic and metabolomic analyses reveal resistance mechanisms in Chilli pepper roots of resistant and susceptible varieties to *Phytophthora capsici* infection

**DOI:** 10.3389/fpls.2025.1638114

**Published:** 2025-10-20

**Authors:** Zhou Heng, Xiaowan Xu, Tao Li, Xiaomei Xu

**Affiliations:** Vegetable Research Institute, Guangdong Academy of Agriculture Sciences/Guangdong Key Laboratory of New Technology Research of Vegetables, Guangzhou, China

**Keywords:** Chilli pepper, proteomic, metabolomic, resistance mechanisms, *Phytophthora capsici*

## Abstract

**Introduction:**

Phytophthora blight, caused by *Phytophthora capsici*, poses a severe threat to global pepper production.

**Methods:**

This study systematically investigated resistance mechanisms in the root of blight-resistant pepper cultivar CM334 compared to the susceptible genotype NMCA10399 using integrated proteomic and metabolomic analyses at 0, 12, and 36 hour post-inoculation.

**Results:**

The results showed that arachidonic acid (AA) was the primary differential metabolite between the resistant and susceptible varieties, while the ABC transporter pathway was the main differential protein pathway. The relative content of salicylic acid (SA) showed opposite trends in the early stages of infection in the two varieties. In the resistant variety, proteins involved in plant–pathogen interaction pathways, such as NHO1, Rd19, WRKY1, and WRKY2, were upregulated.

**Discussion:**

This study characterized the differences in metabolite and protein expression profiles between resistant and susceptible pepper varieties after inoculation, identified potential key metabolites and proteins, and provided new theoretical support for the study of pepper blight resistance mechanisms and the breeding of resistant varieties.

## Introduction

Chilli pepper (*Capsicum annuum*) is one of the most economically and agriculturally important crops, used as a vegetable, spice, food coloring agent, and medicinal material (Liu et al., 2024). Global annual pepper production reaches approximately 38 million tons (FAO, 2023). Pepper blight, caused by the oomycete pathogen *Phytophthora capsici*, is a devastating disease in global pepper production areas and causes more than $100 million in losses annually (Bosland, 2008; Li et al., 2020). Initially described by L.H. Leonian in 1922 as a pathogen of peppers in New Mexico, USA, it is now widely distributed in temperate and tropical countries (Quesada-Ocampo et al., 2023; Leonian, 1922). As a soil-borne pathogen, it can infect almost all parts of the pepper plant, causing various symptoms, including root rot, stem base rot, and leaf and fruit blight (Yuan et al., 2024). Unfortunately, current management strategies, including agricultural practices, chemical applications, and planting resistant varieties, have not effectively prevented this disease (Garcés-Fiallos et al., 2025; Kaur et al., 2024). Although pesticide control efficacy ranges from 14% to 100% under specific experimental conditions, considering pathogen resistance in practical production and environmental sustainability, the development of more efficient and targeted control measures remains imperative (Wan and Liew, 2020). Therefore, studying the resistance mechanisms of peppers against *P. capsici* is crucial for improving pepper management and resistance breeding programs.

Numerous studies on plant resistance mechanisms have shown that pattern recognition receptors (PRRs) in plants interact with various pathogen-associated molecular patterns (PAMPs), triggering PAMP-triggered immunity (PTI) (Hind et al., 2016; Morris and Moury, 2019; Dong and Ronald, 2019),. During infection, PTI can be suppressed by effector proteins encoded by pathogens. These effectors are recognized by plant-encoded nucleotide-binding site leucine-rich repeat (NBS-LRR) resistance (R) genes, leading to effector-triggered immunity (ETI), which often induces a form of programmed cell death known as the hypersensitive response (HR) (Mathieu et al., 2014; Morris and Moury, 2019; Dong and Ronald, 2019).

To effectively manage blight, resistant resources have been screened (Mohammadbagheri et al., 2021, 2022), and natural or genetic populations with different resistance traits have been used to locate resistance-associated loci (Kaur et al., 2024; Lozada et al., 2021). Siddique et al (Siddique et al., 2019). identified three major-effect quantitative trait loci (QTLs) on pepper chromosome P5 (5.1, 5.2, and 5.3), which exhibited broad-spectrum resistance to three *P. capsici* strains. Additionally, QTLs with epistatic interactions and small effects were detected on other chromosomes. Lozada et al (Lozada et al., 2021). identified major-effect QTLs associated with resistance to *P. capsici* root rot on chromosomes P5, P8, and P9 of pepper based on a recombinant inbred line (RIL) population derived from the hybridization between ‘CM334’ and ‘Early Jalapeno’. These QTLs explained 19.7% to 30.4% of the phenotypic variation in resistance. Zhang et al (Zhang et al., 2023), through genome-wide association study (GWAS) analysis, located *P. capsici* resistance loci to a 1.68 Mb interval on chromosome 5, containing nine genes, with Capana05g000704 encoding a leucine-rich repeat receptor-like serine/threonin protein kinase being the most likely candidate gene for *P. capsici* resistance. Using different germplasm panels with limited overlap with Zhang’s work, Kaur et al (Kaur et al., 2024). identified 330 single nucleotide polymorphism (SNP) markers significantly associated with resistance, distributed across all 12 chromosomes, indicating a complex genetic basis for pepper resistance to *P. capsici*. Yuan et al (Yuan et al., 2024), through GWAS, identified two major resistance loci on chromosomes 5 (CaRPc5.1) and 10 (CaRPc10.1). In summary, different resistance materials have been located to different loci, and it is difficult to find a single major-effect QTL, indicating that the resistance mechanism to blight is highly complex.

To further reveal the molecular resistance mechanisms at the molecular level, single-omics or multi-omics approaches have been used to analyze differences in various resistant materials during the infection process. Shi et al (Shi et al., 2024), through iTRAQ-based proteomic analysis, found that differentially expressed proteins (DEPs) were significantly enriched in secondary metabolite biosynthesis, carbon fixation in photosynthesis, and pyruvate metabolism pathways. Li et al (Li et al., 2024), through analysis of mRNA and miRNA, revealed the regulatory network of miRNAs and target genes in peppers infected with *P. capsici*. Li et al (Li et al., 2020), through dynamic transcriptome analysis of pepper whole roots, revealed that pepper roots enhance resistance to *P. capsici* by activating the phenylpropanoid biosynthesis pathway. Lei et al (Lei et al., 2023), through combined metabolomic and transcriptomic analysis, found that the flavonoid biosynthesis pathway plays an important role in pepper resistance to *P. capsici*.

It is well known that changes at the mRNA level do not always directly reflect protein expression levels, as mRNA translation efficiency and protein stability can affect the final protein levels (Buccitelli and Selbach, 2020). Direct study of protein expression and modification can more directly reflect the functional state of cells. However, no studies have combined proteomics with metabolomics to analyze the resistance mechanisms of pepper blight. To investigate the changes in pepper roots following infection by *P. capsici* at both proteomic and metabolomic levels, this study used metabolomics combined with proteomics to analyze the roots of resistant and susceptible pepper varieties at 0, 12, and 36 hour post-inoculation with *P. capsici*. The study identified key differential metabolites and proteins, with a focus on differential proteins in plant–pathogen interaction pathways, and validated these proteins using qPCR. This study provides new insights into the molecular mechanisms of pepper resistance to blight and offers a new theoretical direction for breeding resistant varieties.

## Materials and methods

### Plant and pathogen materials

The study selected two pepper accessions: CM334 (hereinafter referred to as R), a landrace from Mexico renowned for its exceptional resistance to *P. capsici*, and NMCA10399 (hereinafter referred to as S), which is highly susceptible to *P. capsici*, as evidenced by its vulnerability to all tested *P. capsici* isolates. Plant materials were cultivated in plastic pots (20 cm diameter) containing plant growth substrate (Floragard, Germany) and grown under controlled conditions at 26 ± 2°C with 70% relative humidity and a 14-h-light/10-h-dark photoperiod. When the plants reached the six-leaf stage, they were inoculated with the *P. capsici* isolate Byl4, which was originally isolated from infected pepper plants in Baiyun field, Guangzhou, Guangdong Province, China (Xu et al., 2016). Twelve hours prior to inoculation, the plants were thoroughly watered. Subsequently, 2 ml of Byl4 spore suspension, at a concentration of 5 × 10^4^ zoospores/ml, was injected into the root-shoot soil line, following the method described by Xu et al (Xu et al., 2016). with minor modifications. In brief, The preserved Phytophthora capsici (Byl4) isolate was reactivated and cultured on 20% V8 agar medium(200 mL V8 juice, 3.0 g CaCO_4_, 20 g agar, 800 mL distilled water, pH 6.3). Initially, it was incubated in the dark at 25°C for 3 days, followed by 3–4 days under a 12-hour light/12-hour dark cycle. Subsequently, 10 mL of sterile water was added to the culture dish, which was then placed in a refrigerator at 4°C for 30 minutes and allowed to stand at room temperature (23–25°C) for an additional 30 minutes to induce spore release. The spore suspension was collected, and the number of motile spores was quantified using a hemocytometer. The suspension was then adjusted to the required concentration for experimental use. The inoculated plants were then continued to be grown under the same environmental conditions.

Referencing our group’s published transcriptomic study (Li et al., 2020), the experiment included two genotypes (susceptible ‘S’ and resistant ‘R’) across three time points (0h, 12h, 36h), designated as S0, S12, S36, R0, R12, R36. Each designation comprised three biological replicates. To meet the sample requirements for both metabolomic and proteomic analyses, roots from four pepper seedlings were pooled per biological replicate. The harvested roots were subsequently ground into powder using a mortar and pestle under liquid nitrogen.

### Metabolite extraction, detection, data processing, and annotation

#### Sample preparation and extraction

Samples were subjected to freeze-drying using a vacuum freeze-dryer (Scientz-100F). The freeze-dried samples were then crushed in a mixer mill (MM 400, Retsch) with a zirconia bead for 1.5 minutes at a frequency of 30 Hz. For extraction, 100 mg of the lyophilized powder was dissolved in 1.2 mL of a 70% methanol solution. The mixture was vortexed for 30 seconds every 30 minutes, repeated six times in total, and then left in a refrigerator at 4°C overnight. After centrifugation at 12,000 rpm for 10 minutes, the supernatant was filtered through a 0.22 μm pore size filter (SCAA-104, ANPEL, Shanghai, China) before being subjected to UPLC-MS/MS analysis.

#### UPLC-MS/MS analysis

The sample extracts were analyzed using a UPLC-ESI-MS/MS system, which consisted of a UPLC (SHIMADZU Nexera X2) and an MS (Applied Biosystems 4500 Q TRAP). The analytical conditions were as follows: The UPLC was equipped with an Agilent SB-C18 column (1.8 µm, 2.1 mm × 100 mm). The mobile phase was composed of solvent A (pure water with 0.1% formic acid) and solvent B (acetonitrile with 0.1% formic acid). The gradient program started with 95% A and 5% B, linearly changed to 5% A and 95% B within 9 minutes, held at 5% A and 95% B for 1 minute, then adjusted back to 95% A and 5% B within 1.1 minutes and maintained for 2.9 minutes. The flow rate was set at 0.35 mL/min, the column oven temperature was 40°C, and the injection volume was 4 μL. The eluent was directed to an ESI-triple quadrupole-linear ion trap (QTRAP)-MS.

The mass spectrometer (AB4500 Q TRAP UPLC/MS/MS System) was equipped with an ESI Turbo Ion-Spray interface and operated in both positive and negative ion modes, controlled by Analyst 1.6.3 software (AB Sciex). The ESI source parameters were set as follows: ion source, turbo spray; source temperature, 550°C; ion spray voltage (IS), 5500 V (positive ion mode) or -4500 V (negative ion mode); ion source gas I (GSI), gas II (GSII), and curtain gas (CUR) were set at 50, 60, and 25.0 psi, respectively; collision-activated dissociation (CAD) was set to high. Instrument tuning and mass calibration were performed using 10 and 100 μmol/L polypropylene glycol solutions in QQQ and LIT modes, respectively. QQQ scans were acquired as MRM experiments with collision gas (nitrogen) set to medium. DP and CE for individual MRM transitions were optimized accordingly. Specific MRM transitions were monitored for each period based on the elution profile of the metabolites.

#### Data analysis

Unsupervised principal component analysis (PCA) was performed using the prcomp function in R (https://www.r-project.org). The data were scaled to unit variance before PCA. Hierarchical cluster analysis (HCA) results for samples and metabolites were visualized as heatmaps with dendrograms, while Pearson correlation coefficients (PCC) between samples were calculated using the cor function in R and presented as heatmaps. Both HCA and PCC were conducted using the R package pheatmap. For HCA, normalized signal intensities of metabolites (unit variance scaling) were displayed as a color spectrum. Significantly regulated metabolites between groups were identified based on VIP values ≥ 1 and absolute log2 fold changes ≥ 1. VIP values were extracted from OPLS-DA results, which included score plots and permutation plots, generated using the R package MetaboAnalystR. The data were log2-transformed and mean-centered before OPLS-DA. To prevent overfitting, a permutation test (200 permutations) was performed. Identified metabolites were annotated using the KEGG Compound database and mapped to the KEGG Pathway database. Pathways with significantly regulated metabolites were subjected to metabolite set enrichment analysis (MSEA), and their significance was determined by hypergeometric test p-values.

### Protein extraction, digestion, and LC–MS/MS analysis

#### Sample preparation and processing

The samples were homogenized in a lysis buffer containing 2.5% SDS and 100 mM Tris-HCl (pH 8.0). Subsequently, the samples underwent ultrasonication treatment. After centrifugation, proteins in the supernatant were precipitated by adding four times the volume of pre-cooled acetone. The resulting protein pellets were dissolved in a solution of 8 M urea and 100 mM Tris-Cl. Following another round of centrifugation, the supernatant was used for a reduction reaction with 10 mM DTT at 37°C for 1 hour, followed by an alkylation reaction with 40 mM iodoacetamide at room temperature in the dark for 30 minutes. The protein concentration was then measured using the Bradford method. The urea concentration was diluted to below 2 M using 100 mM Tris-HCl (pH 8.0). Trypsin was added at an enzyme-to-protein ratio of 1:50 (w/w) for overnight digestion at 37°C. The next day, TFA was used to adjust the pH to 6.0 to terminate the digestion. After centrifugation at 12,000×g for 15 minutes, the supernatant was subjected to peptide purification using a Sep-Pak C18 desalting column. The eluate was vacuum-dried and stored at -20°C for later use.

#### TMT labeling and fractionation

TMT labeling was performed according to the manufacturer’s instructions. Peptides were reconstituted in TMT reagent buffer and labeled with different TMT labeling reagents. The labeled samples were then mixed and subjected to Sep-Pak C18 desalting. The complex mixture was fractionated using high pH reverse phase chromatography and combined into 15 fractions. Each fraction was vacuum-dried and stored at -80°C until MS analysis.

#### LC-MS/MS analysis

LC-MS/MS data acquisition was performed on an Orbitrap Exploris 480 mass spectrometer coupled with an Easy-nLC 1200 system. Peptides were loaded via an auto-sampler and separated on a C18 analytical column (75 μm × 25 cm, C18, 1.9 μm, 100 Å). The mobile phase consisted of solvent A (0.1% formic acid) and solvent B (80% ACN, 0.1% formic acid) to establish the separation gradient. A constant flow rate of 300 nL/min was maintained. For DDA mode analysis, each scan cycle included one full-scan mass spectrum (R = 60 K, AGC = 300%, max IT = 20 ms, scan range = 350–1500 m/z) followed by 20 MS/MS events (R = 15 K, AGC = 100%, max IT = auto, cycle time = 2 s, TurboTMT enabled). The HCD collision energy was set to 35, with an isolation window for precursor selection of 1.2 Da. Former target ion exclusion was enabled for 35 seconds.

### Protein identification and quantification

The MS raw data were processed using MaxQuant (V1.6.6) and the Andromeda database search algorithm. The spectra files were compared with the UniProt proteome database (Capsicum_annuum, https://www.uniprot.org/proteomes/UP000222542) under the following settings: TMT quantification mode was enabled; Variable modifications included Oxidation (M), Acetyl (Protein N-term), and Deamidation (NQ); Fixed modifications were set as Carbamidomethyl (C); Trypsin/P was chosen for digestion; For the MS1 match tolerance, it was initially set at 20 ppm and then adjusted to 4.5 ppm for the main search, while the MS2 tolerance was maintained at 20 ppm. The search outcomes were filtered with a 1% FDR threshold at both the protein and peptide levels. Proteins identified as decoy hits, contaminants, or those solely recognized by sites were excluded. The remaining identifications were utilized for subsequent quantification analysis.

### Integrated analysis of proteomics and metabolomics data

Based on the differential metabolite analysis results of this experiment, combined with the differential protein analysis results, the differential proteins and differential metabolites of the same group were simultaneously mapped onto the KEGG pathway map.

### RNA extraction and quantitative real-time PCR

Total RNA was extracted from frozen root powder using the Column Plant RNAout Kit (TIANDZ, Beijing, China). Subsequently, cDNA was synthesized with the PrimeScript™ RT reagent Kit with gDNA Eraser (Takara, Dalian, China). Three biological replicates were prepared for each genotype, and each biological replicate consisted of three technical replicates.The PCR reactions were conducted in a 20-μl reaction volume, comprising 1 μl of primer (10 μM), 1 μl of cDNA, 8 μl of PCR-grade water, and 10 μl of AceQ qPCR SYBR Green Master Mix (Vazyme, Nanjing, China), using a Roche LightCycler 480 Q-PCR system (Roche, Basel, Switzerland).The PCR protocol began with an initial denaturation step at 95 °C for 5 min, followed by 45 cycles of 95 °C for 10 s and 60 °C for 30 s. After each run, a melting curve analysis was performed to confirm the specificity of the amplified products. The primer sequences and reference gene information used for qPCR are detailed in **Supplementary Table S1**.

## Results

### Pepper metabolome profiling in response to *P. capsici* infecting

**Figure 1:**
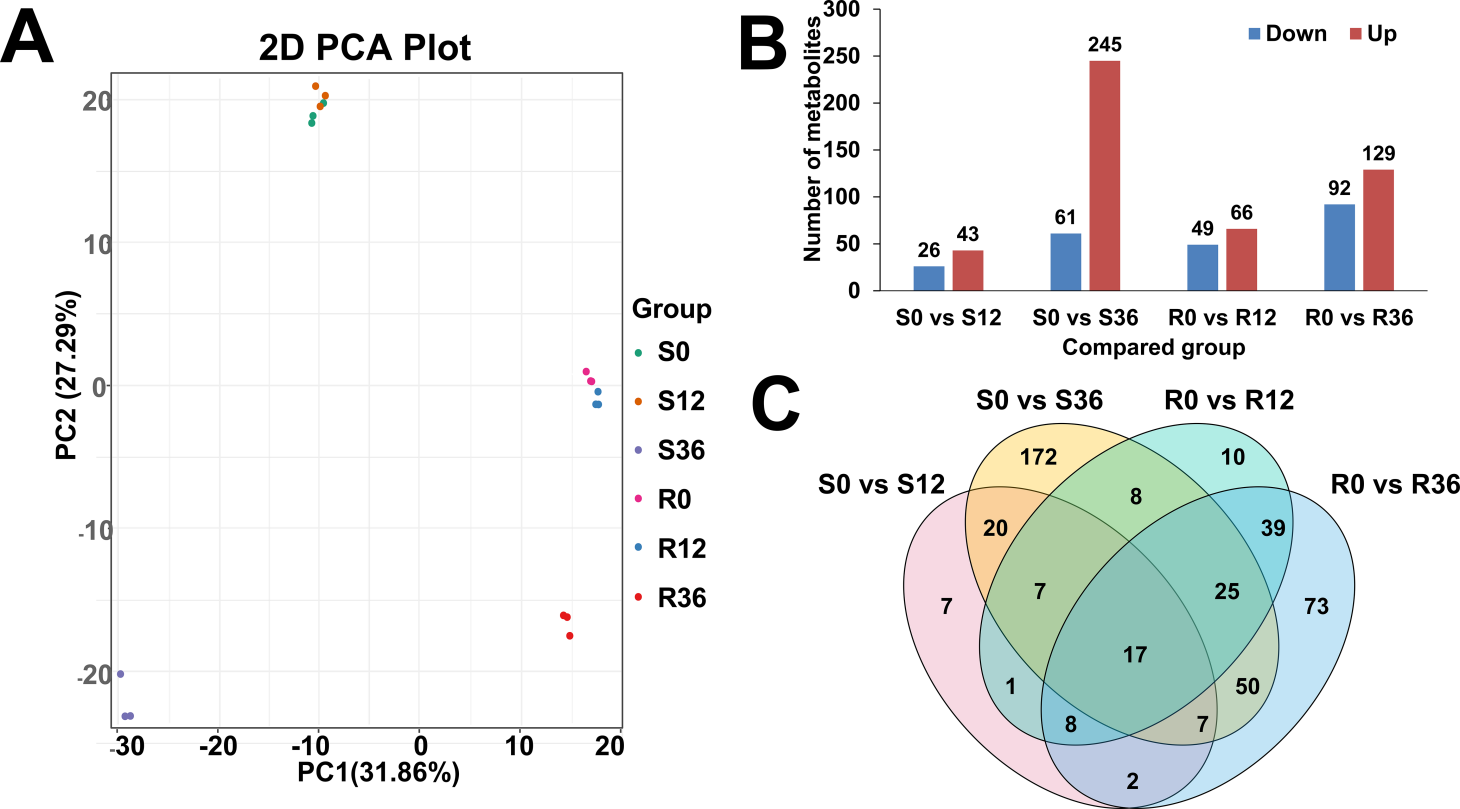
Overview of metabolome data. **(A)** PCA plot of metabolome result. **(B)** Bar plot of different expressed metabolites(DEM). **(C)** Venn plot of DEM.

Comprehensive metabolomic profiling identified 997 metabolites across experimental groups, with lipid species (n=175), alkaloids (n=150), and phenolic acids (n=143) constituting the predominant chemical classes (**Supplementary Table S2**). Principal component analysis (PCA) revealed distinct temporal dynamics (**Figure 1A**): S0 and S12 formed a tight cluster indicative of early-stage metabolic stability in susceptible genotypes, while R0 and R12 grouped separately, suggesting genotype-specific reprogramming during initial defense activation. Notably, S36 and R36 exhibited spatial divergence from earlier timepoints, implying progressive metabolic divergence between resistant and susceptible lines under prolonged pathogen pressure.

Differential expression analysis (FDR-corrected p<0.05, |log2FC|≥1) identified 69, 306, 115, and 221 differentially abundant metabolites (DAMs) in S0 vs. S12, S0 vs. S36, R0 vs. R12, and R0 vs. R36 comparisons, respectively (**Figure 1B**). The upregulated: downregulated ratios (26:43, 61:245, 49:66, 92:129) demonstrated asymmetric metabolic reprogramming, with susceptible genotypes showing pronounced downregulation (245/306 metabolites) at S36, potentially reflecting resource reallocation or catabolic dominance during late infection. Venn diagram results showed that the four comparison groups had 7, 172,10, and 73 unique metabolites, respectively (**Figure 1C**).

### Pepper proteome profiling in response to *P. capsici* infecting

**Figure 2:**
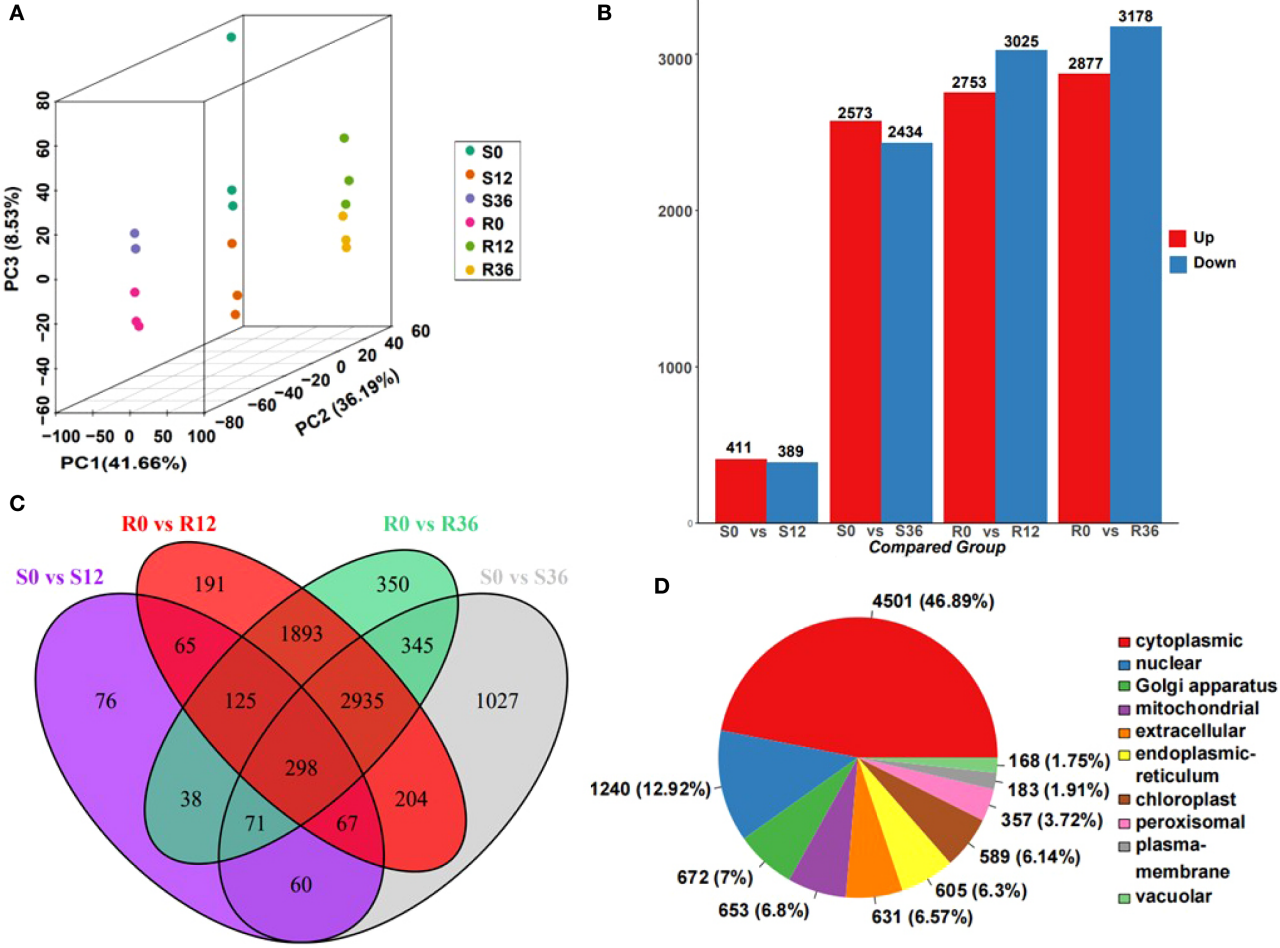
Overview of proteome data. **(A)** PCA plot of proteome result. **(B)** Bar plot of different expressed protein(DEP). **(C)** Venn plot of DEP. **(D)** Distribution of identified proteins from the proteome of chili pepper into Subcellular localization.

Proteomic profiling identified 9,599 proteins (**Supplementary Table S3**), with principal component analysis (PCA) demonstrating robust clustering of biological replicates across experimental groups. The PCA dimensions revealed distinct temporal dynamics: S0 and S12 clustered closely, as did R12 and R36, while S36 and R0 formed a separate group, suggesting stage-specific molecular reprogramming during pathogen interaction (**Figure 2A**). Differential expression analysis identified 800 (411 upregulated, 389 downregulated), 5,007 (2,573↑, 2,434↓), 5,778 (2,753↑, 3,025↓), and 6,055 (2,877↑, 3,178↓) significantly altered proteins in the S0 vs. S12, S0 vs. S36, R0 vs. R12, and R0 vs. R36 comparisons, respectively (**Figure 2B**, **Supplementary Table S4**). Venn diagram analysis highlighted 76 unique proteins in the susceptible genotype (S0 vs. S12) compared to 191 in the resistant genotype (R0 vs. R12) (**Figure 2C**), indicating genotype-specific proteomic remodeling during early infection.

Subcellular localization predictions revealed cytoplasmic dominance (46.89%), followed by nuclear (12.92%) and Golgi apparatus distributions (**Figure 2D**). Notably, differentially abundant proteins in S0 vs. S12 were primarily localized to the cytoplasm, extracellular space, chloroplasts, endoplasmic reticulum, nucleus, and mitochondria. In contrast, other comparisons showed enrichment in cytoplasmic, nuclear, mitochondrial, Golgi, chloroplast, and extracellular compartments (**Supplementary Figure S1**). The pronounced extracellular proteome alterations in the susceptible genotype at 12 hours post-inoculation (hpi) suggest a critical window for pathogen recognition evasion or failed defense signaling, potentially explaining susceptibility mechanisms.

### Combined proteomic and metabolomic analysis

**Figure 3:**
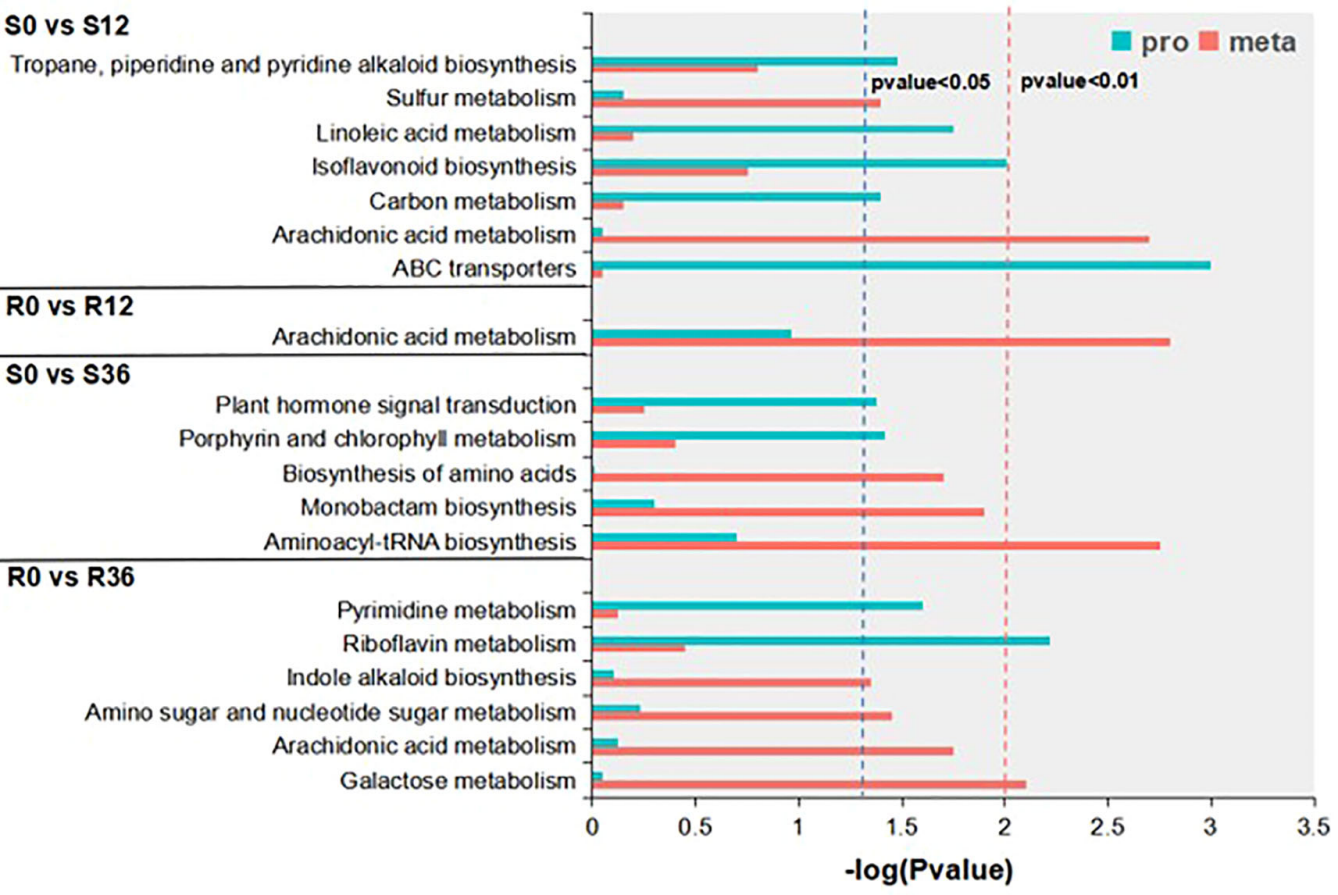
KEGG enrichment results of conjoint analysis.

Integrated proteomic-metabolomic analysis revealed distinct pathway dynamics between resistant (R) and susceptible (S) genotypes during *P. capsici* infection. KEGG enrichment of differentially abundant proteins (DAPs) and metabolites (DAMs) was conducted under dual significance thresholds (p<0.05 and p<0.01) (**Figure 3**). At the p < 0.05 level, DAPs in S0 vs S12 were enriched in the Tropane, piperidine and pyridine alkaloid biosynthesis pathway, linoleic acid metabolism pathway, isoflavonoid biosynthesis pathway, carbon metabolism pathway, and ABC transporters pathway. DAMs were enriched in the sulfur metabolism pathway. DAMs in R0 vs R12 were enriched in the arachidonic acid metabolism pathway, with no enriched protein pathways. DAPs in S0 vs S36 were enriched in the plant hormone signal transduction pathway and porphyrin and chlorophyll metabolism pathway. DAMs were enriched in the biosynthesis of amino acids pathway, monobactam biosynthesis pathway, and aminoacyl-tRNA biosynthesis pathway. DAPs in R0 vs R36 were enriched in the pyrimidine metabolism pathway and riboflavin metabolism pathway. DAMs were enriched in the amino sugar and nucleotide sugar metabolism pathway, arachidonic acid metabolism pathway, and galactose metabolism pathway. At the p-value < 0.01 level, DAMs in both S0 vs. S12 and R0 vs. R12 were enriched in the arachidonic acid metabolism pathway, and S0 vs S12 was also enriched in the ABC transporters protein pathway. S0 vs S36 was enriched in the aminoacyl-tRNA biosynthesis metabolic pathway, and R0 vs R36 was enriched in the galactose metabolism metabolic pathway and riboflavin metabolism protein pathway. None of these combined analysis results showed a pathway that was simultaneously significantly enriched for both DAPs and DAMs. Based on these results, we speculate that the arachidonic acid metabolism pathway and the ABC transporters pathway may be the key pathway related to *P. capsici* resistance.

### In-depth analysis of metabolomics data

To further elucidate the changes in arachidonic acid (AA) within the materials, we conducted an in-depth analysis of the KEGG enrichment results derived from the metabolomics dataset. The number of significantly enriched KEGG pathways (P < 0.05) across the four experimental groups were 2, 2, 3, and 3, respectively (**Supplementary Table S5**). Specifically, the pathways identified were as follows: for the S0 vs S12 comparison, the enriched pathways included arachidonic acid metabolism and sulfur metabolism; for S0 vs S36, the pathways were aminoacyl-tRNA biosynthesis, monobactam biosynthesis, and biosynthesis of amino acids; in the R0 vs R12 comparison, the pathways were arachidonic acid metabolism and flavone and flavonol biosynthesis; and for R0 vs R36, the enriched pathways included galactose metabolism, arachidonic acid metabolism, and amino sugar and nucleotide sugar metabolism.

**Figure 4:**
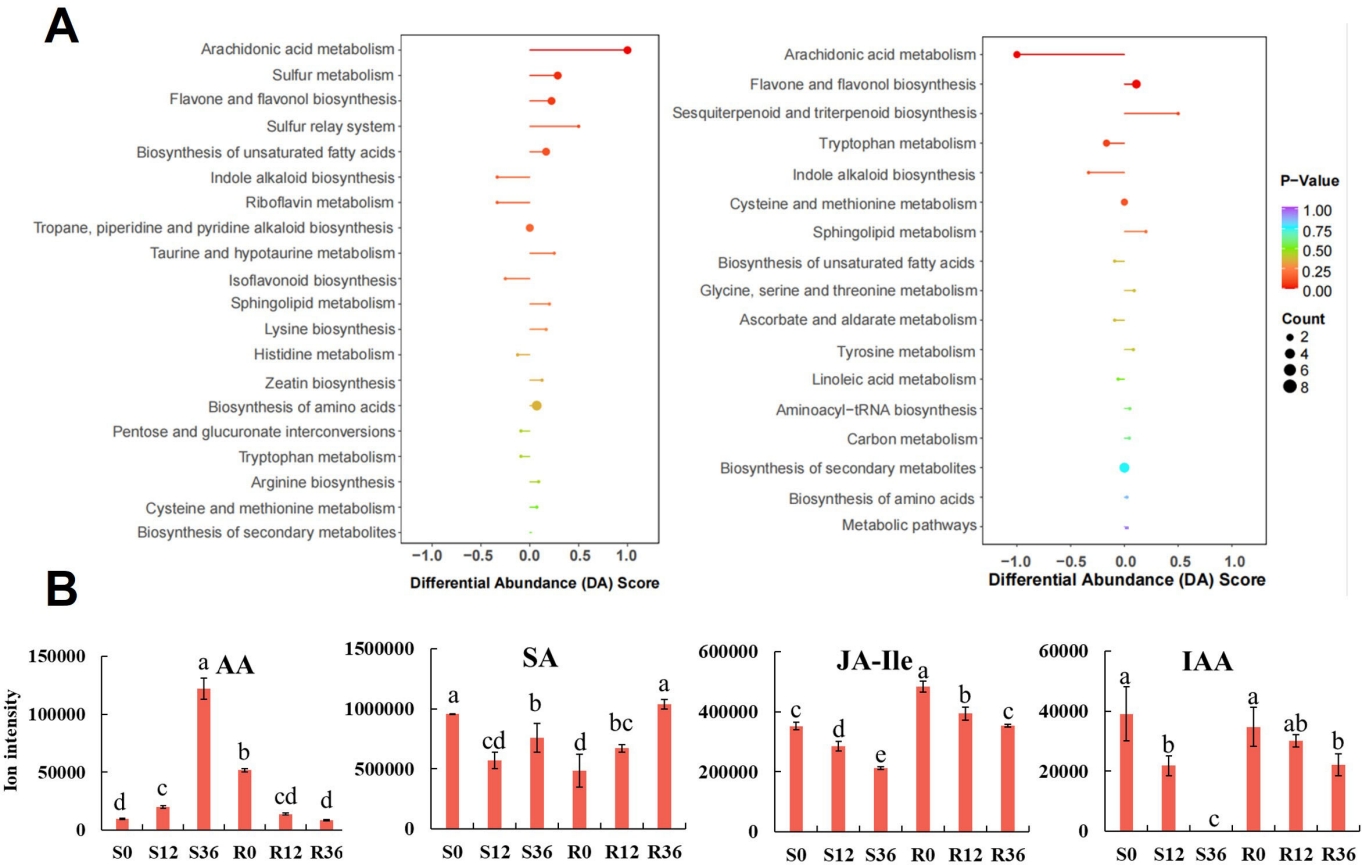
KEGG enrichment results of metabolomic analysis **(A)** and Bar graph of AA and phytohormone ion intensity in metabolome **(B)**.

Notably, the arachidonic acid metabolism pathway exhibited a distinct regulatory pattern, being significantly upregulated in the susceptible variety (S) while being downregulated in the resistant variety (R) (**Figure 4A**). Further investigation into this pathway revealed that arachidonic acid itself was the primary differential metabolite (**Figure 4B**). At the initial time point (0 h), the relative content of arachidonic acid was lower in the susceptible variety (S) compared to the resistant variety (R). However, during the infection process, the levels of arachidonic acid increased in S, whereas they decreased in R (**Figure 4B**). These findings indicate that arachidonic acid represents the most significant differential metabolite distinguishing resistant and susceptible varieties, highlighting its potential role in the differential responses to infection. During the plant resistance process, changes in hormone levels occur. Metabolomics data showed that Salicylic acid (SA)in S decreased initially upon infection, while it increased in R (**Figure 4B**). Jasmonic acid isoleucine (JA-Ile) is highly correlated with jasmonic acid (JA) (Shah, 2005). The initial relative content of JA-Ile was lower in S than in R, but both decreased during the infection process (**Figure 4B**). Indole-3-acetic acid (IAA) decreased in both S and R, and interestingly, it was almost undetectable in S at 36 h (**Figure 4B**). This result may indicate that root growth was completely halted due to pathogen infection.

### In-depth analysis of proteomics data

KEGG analysis of the proteomics data revealed that the number of significantly enriched pathways in the comparisons S0 vs S12, S0 vs S36, R0 vs R12, and R0 vs R36 were 36, 11, 16, and 12, respectively (**Supplementary Table S6**). Among these, the differential proteins in S0 vs S12 were predominantly enriched in pathways such as chemical carcinogenesis, drug metabolism-cytochrome P450, and xenobiotic metabolism by cytochrome P450, which also included the plant–pathogen interaction pathway. While the plant–pathogen interaction pathway was similarly enriched in R0 vs R12, it did not reach statistical significance. Notably, the plant–pathogen interaction pathway contained 235 differential proteins in the resistant variety (R), compared to only 44 in the susceptible variety (S).

**Figure 5:**
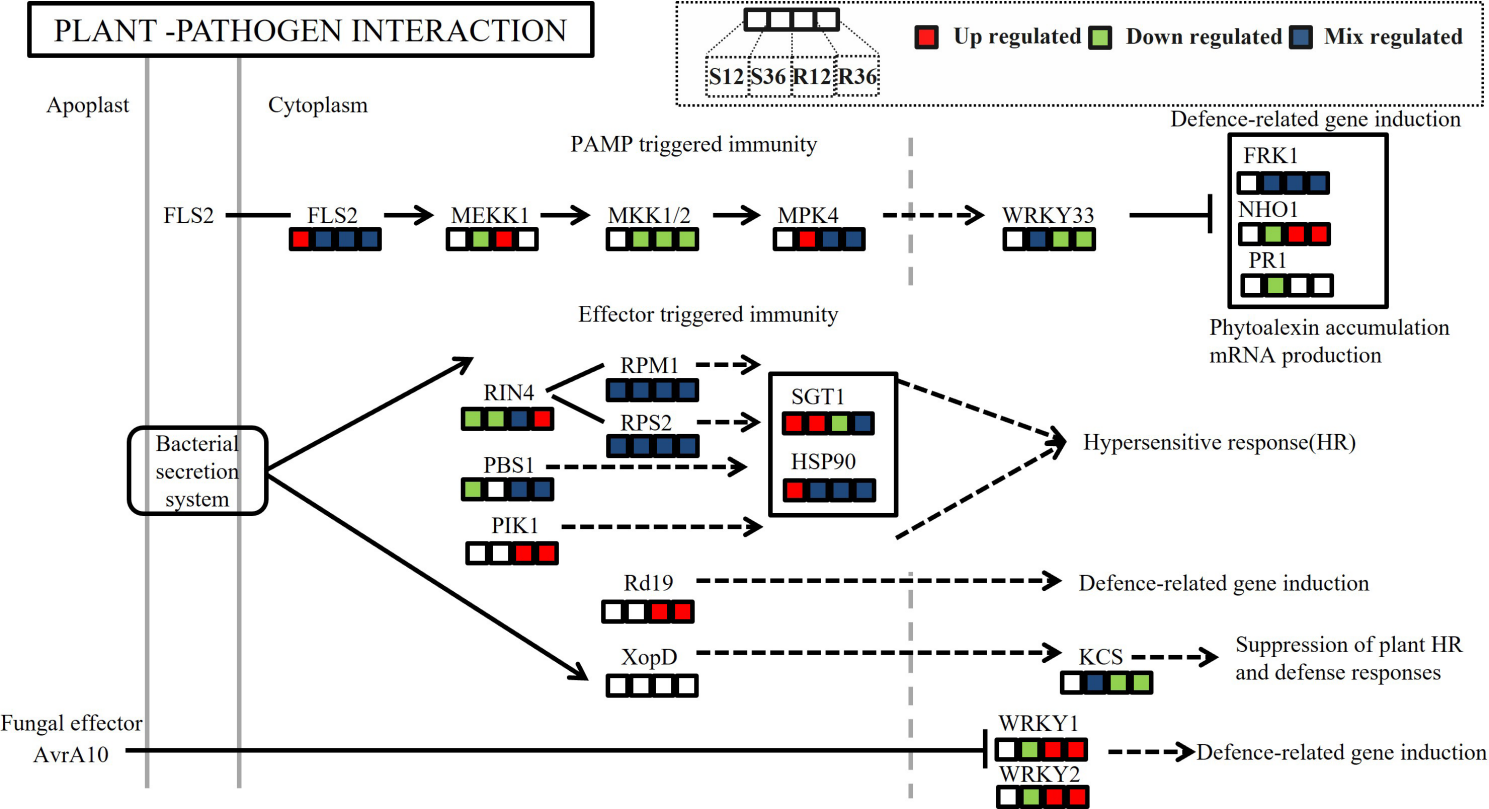
Differences in protein expression abundance in the KEGG pathway plant-pathogen interaction across different experimental groups. (In the figure, the colored blocks from left to right represent the changes in protein expression in the four comparison groups: S12 vs S0, S36 vs S0, R12 vs R0, and R36 vs R0.Red indicates significant upregulation of protein expression at the site, green indicates downregulation, and blue indicates a mix of upregulation and downregulation at the site).

The plant–pathogen interaction pathway is a critical pathway reflecting plant defense mechanisms. In our analysis of differential proteins within this pathway, we observed distinct differences in both PAMP-triggered immunity (PTI) and effector-triggered immunity (ETI) processes in the resistant variety (**Figure 5**). In the PTI pathway, WRKY33, a transcription factor downstream of the MAPK signaling pathway that suppresses the expression of resistance genes, was significantly downregulated in the resistant variety. This downregulation led to the upregulation of NHO1, a protein that promotes resistance gene expression. In the ETI pathway, the cytoplasmic protein Rd19, which is induced by bacterial effector proteins, was upregulated in R, thereby enhancing the expression of defense-related genes. Additionally, WRKY1 and WRKY2, which are known to induce defense gene expression, were also upregulated in R. These findings collectively highlight the differential regulation of key immune responses in resistant and susceptible varieties.

The ABC transporters pathway, similar to the plant–pathogen interaction pathway, was not significantly enriched in R; however, a greater number of proteins were detected in R than in S. This observation suggests that R may exhibit a more timely response to pathogen challenge, potentially leading to a higher number of detectable proteins. Given that the ABC transporters pathway was the only pathway significantly enriched at the 0.01 level in the combined analysis during the initial infection stage, we conducted a detailed investigation of the proteins within this pathway. A total of 54 ABC transporter proteins were identified, including the pleiotropic drug resistance protein 2 (PDR)(**Supplementary Table S7**). The expression levels of PDR were significantly lower in S0 and S12 compared to R0 and R12. Previous studies have reported that the interaction of PDR with LecRK influences resistance in pepper to *P. capsica* (Wang et al., 2015).

**Figure 6:**
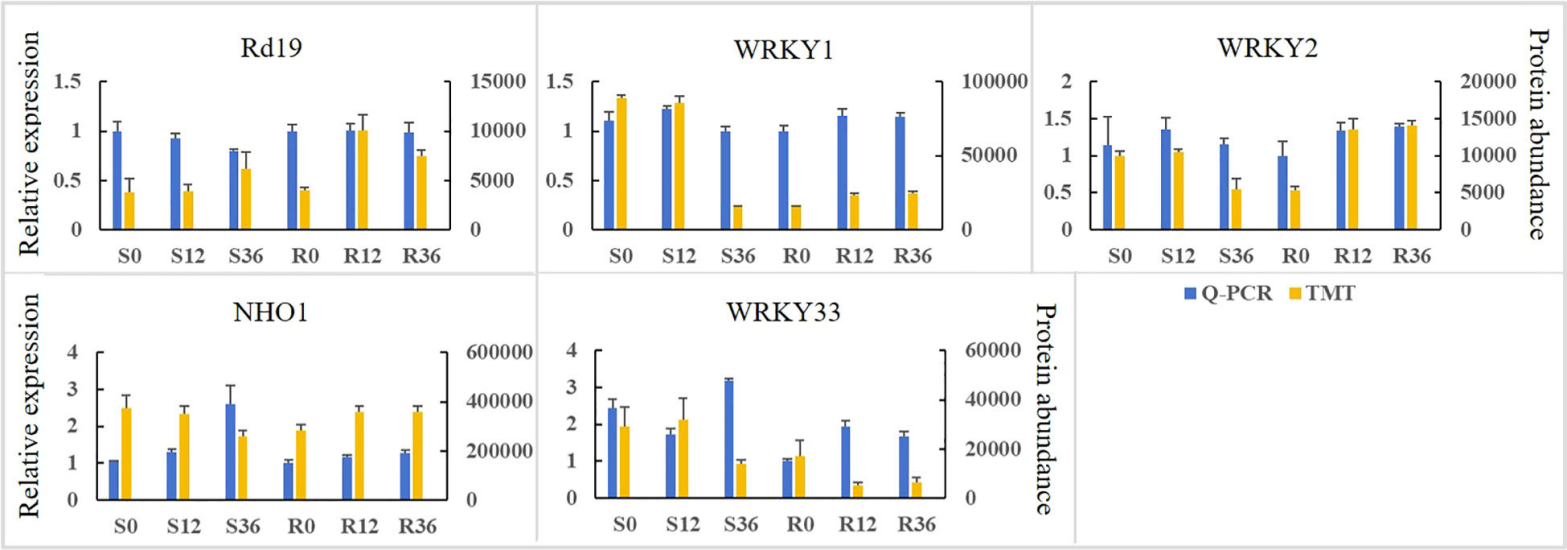
The expression abundance of proteins associated with disease resistance and the relative expression levels of their encoding genes in the pathogen-plant interaction pathway.

To validate the expression abundance of key disease resistance-related proteins, we quantified the relative transcript levels of their encoding genes. As shown in **Figure 6**, a positive correlation was observed between protein abundance and mRNA expression levels for only WRKY1 and 2. The gene expression levels of NHO1 and their protein abundance showed a consistent trend only in the resistant cultivar. The relative gene expression levels of A and B and their protein abundance failed to show a consistent trend. This discrepancy may be attributed to post-translational modifications or other regulatory factors.

## Discussion

Pepper, an important vegetable and spice crop, is severely threatened by Phytophthora blight, a major disease that poses a significant challenge to the pepper industry (Admassie et al., 2023). Understanding the molecular mechanisms underlying pepper’s resistance to blight is critical for developing environmentally friendly and efficient disease control strategies and for breeding blight-resistant pepper varieties (Bagheri et al., 2020). While previous studies have identified resistance loci and markers associated with resistance (Zhang et al., 2023; Yuan et al., 2024; Siddique et al., 2019; Mohammadbagheri et al., 2022), there is a notable lack of multi-omics data characterizing the interactions between resistant and susceptible varieties and the pathogen. In this study, we employed proteomics and metabolomics to analyze resistant and susceptible pepper materials before and after inoculation with the blight pathogen. Our analysis identified several metabolites and proteins associated with resistance, providing valuable insights into the mechanisms of pepper’s defense against blight and offering important reference data for future research in this field.

Arachidonic acid (AA), a fatty acid commonly secreted by pathogens during plant infection, serves as an elicitor of plant defense responses to phytopathogens (Dedyukhina et al., 2014; Das, 2018). AA are abundant in the lipids of Phytophthora species and related oomycetes, and are released into plant tissues from spores during the early stages of infection (Teresa et al., 2014). In our study, we observed that after inoculating pepper roots with *P. capsici*, the content of AA in the roots exhibited a negative correlation with resistance. This finding is consistent with a study on anthracnose infection of *Camellia oleifera*, where AA was identified as the main differential metabolite, with higher levels observed in non-inoculated resistant plants (Yang et al., 2022). As previously reported, AA is a major component of pathogen cell membranes but is not commonly found in higher plants (Savchenko et al., 2010). Given that the metabolomics analysis was conducted on pepper roots post-pathogen infection, it is not possible to definitively determine whether the detected AA originated from the pepper plant itself or from *P. capsici*. However, our metabolomics analysis of non-inoculated roots revealed the presence of AA (data not shown), indicating that AA may present in pepper roots. Although sterile culture substrate is utilized, we cannot currently rule out the factor of AA contamination originating from endophytic bacteria within plant tissues. On the other hand, our proteomic data revealed no significant enrichment of AA synthesis-related pathways during the infection process. This finding suggests that pepper plants may lack the capacity for substantial AA production. Taken together, we propose that AA originates primarily from the pathogen rather than the host plant—consistent with its documented role as an elicitor of plant defense responses against phytopathogens (Dedyukhina et al., 2014). In tomato, potato and pepper, exogenous application of AA enhances plant resistance to *P. capsici* (Dye and Bostock, 2021). In summary, AA plays a significant role as a metabolite during pathogen infection of plants. Our results suggest that employing the Host-Induced Gene Silencing (HIGS) strategy to limit the synthesis of AA by the pathogen may represent a promising approach for disease control.

Plant hormones are pivotal in orchestrating plant defense mechanisms against insects and pathogens (Mhlongo et al., 2020). Salicylic acid (SA) and jasmonic acid (JA) are among the most extensively investigated hormones in this context. SA is particularly instrumental in conferring resistance to biotrophic and hemibiotrophic pathogens (Glazebrook, 1999). A hallmark of robust resistance in many studies is the elevation of SA levels following pathogen infection (Zhang et al., 2020; Yang et al., 2023). This hormone is known to elicit the expression of pathogenesis-related (PR) proteins, such as PR1, and to reinforce cell walls, thereby impeding pathogen progression (Sorokan et al., 2023). Exogenous application of SA has been documented to augment pepper’s resistance to *P. capsici* (Yang et al., 2023). Three genes associated with SA biosynthesis were upregulated in the resistant pepper variety upon infection with *P. capsici* (Lei et al., 2023). In the present study, while SA levels declined in the susceptible variety (S) post-infection, they exhibited an increase in the resistant variety (R). This dichotomy suggests the activation of an SA-mediated immune response in R, thereby underscoring SA’s critical role in the resistance of pepper to *P. capsici*.

Jasmonic acid (JA) primarily regulates plant resistance to necrotrophic pathogens (Glazebrook, 1999). However, the relationship between JA and resistance mechanisms against *P. capsici* in peppers remains underexplored. Methyl jasmonate (MeJA), an exogenous activator, has been shown to modestly attenuate symptoms of *P. capsici* infection (Barraza et al., 2022). In this study, although JA itself was not detected, its bioactive form, JA-isoleucine (JA-Ile), was identified. The levels of JA-Ile decreased in both susceptible (S) and resistant (R) varieties following infection, but they remained elevated in R compared to S. This trend aligns with previous findings in studies of *P. capsici*-infected resistant pepper leaves (Ueeda et al., 2005). Additionally, indole-3-acetic acid (IAA) levels decreased in both plant materials, becoming nearly undetectable in S by 36 hours post-infection, suggesting a cessation of root growth in the susceptible variety. Notably, the crosstalk between SA and JA pathways exhibits complexity and context-dependence (Liu et al., 2016). For instance, in pepper, CaASR1 promotes SA- but represses JA-dependent signaling to enhance resistance to bacterial wilt (Huang et al., 2020). The synergistic regulation of SA and JA signaling pathways in conferring resistance to *P.capsici* requires further in-depth investigation.

ABC transporters, also known as ATP-binding cassette transporters, are a class of proteins that rely on the energy generated by ATP hydrolysis to transport substrates across cell membranes (Verrier et al., 2008). They represent the most numerous and functionally diverse class of proteins identified to date (Verrier et al., 2008). *Arabidopsis thaliana* contain 130 ABC transporters (Jasinski et al., 2003). However, only a small fraction of ABC transporters in Arabidopsis have been functionally characterized, and the transport substrates and roles of most members remain unclear. In pepper, Fei et al. identified a novel transporter gene, *CaABCG14*, which regulates the accumulation of capsaicin in pepper septum (Fei et al., 2024). In the present study, the ABC transporter pathway was significantly enriched during the early stages of infection. A total of 54 ABC transporters were identified in pepper roots, including the pleiotropic drug resistance protein (PDR). The expression levels of PDR were significantly lower in the susceptible variety (S0 and S12) compared to the resistant variety (R0 and R12). In Arabidopsis, mutations in PDR have been shown to reduce plant resistance to *P. capsici* (Wang et al., 2015). Based on these findings, we speculate that ABC transporters in pepper play a critical role in conferring resistance to *P. capsici.*

The plant–pathogen interaction (PPI) pathway plays a crucial role during pathogen invasion of plants (Azeez and Adeboye, 2024). Based on our proteomic and metabolomic datasets, we delineated the potential defense network within *Capsicum annuum* against *P. capsici* infection. Numerous studies have reported that proteins in this pathway play significant roles in plant–pathogen interactions (Zhang et al., 2022; Wirthmueller et al., 2013; Peyraud et al., 2017). In this study, we found that in the PAMP-triggered immunity (PTI) pathway, the abundance of the protein encoded by the resistance gene NHO1 was significantly upregulated in the resistant variety. NHO1, a nonhost resistance gene first identified in Arabidopsis, encodes a glycerol kinase that plays a key role in nonhost resistance in plants (Kang et al., 2003). It is involved not only in resistance to bacteria but also to fungi (Lu et al., 2001). In recent years, the NHO1 gene has also been identified in rice, and its overexpression has been shown to enhance resistance to bacterial blight and rice blast (Xiao et al., 2022). These results suggest that a nonhost resistance response occurs in the resistant variety but not in the susceptible variety.

In effector-triggered immunity (ETI), the cytoplasmic protein Rd19, induced by bacterial secreted proteins, was upregulated in the resistant variety (R). Rd19 is a cysteine protease that was first identified in Arabidopsis as playing an important role in ETI against *Ralstonia solanacearum* (Bernoux et al., 2008). In recent years, it has also been found to be important in resistance to powdery mildew (Zeng et al., 2024), suggesting that it may play a role in ETI against multiple pathogens, including blight.

We also found that *WRKY1* and *WRKY2*, which can induce the expression of plant defense genes, were significantly upregulated in R. *WRKY1* plays a key role in resistance to early blight in wild tomatoes by regulating the expression of downstream genes to enhance the plant’s defense response (Shinde et al., 2018). In strawberries, *FaWRKY1* may enhance resistance by activating ROS-dependent defense pathways (Encinas-Villarejo et al., 2009). In apple trees, the *WRKY1* transcription factor enhances resistance to powdery mildew through the regulation of the interconversion of methyl salicylate (MeSA) and salicylic acid (SA) in plant–plant communication (Lan et al., 2024). Overexpression of grape *VqWRKY2* in Arabidopsis enhances resistance to powdery mildew, associated with increased cell death and upregulation of SA signaling pathway-related genes, but not with the JA signaling pathway (Zhang et al., 2024). In pepper, Cheng et al. found that *CaWRKY01–10* and *CaWRKY08–4* can enhance pepper resistance to *P. capsici* by directly binding to resistance genes (Cheng et al., 2024). However, these transcription factors differ from those identified in this study, and the functions of *WRKY1* and *WRKY2* in pepper resistance require further validation.

**Figure 7:**
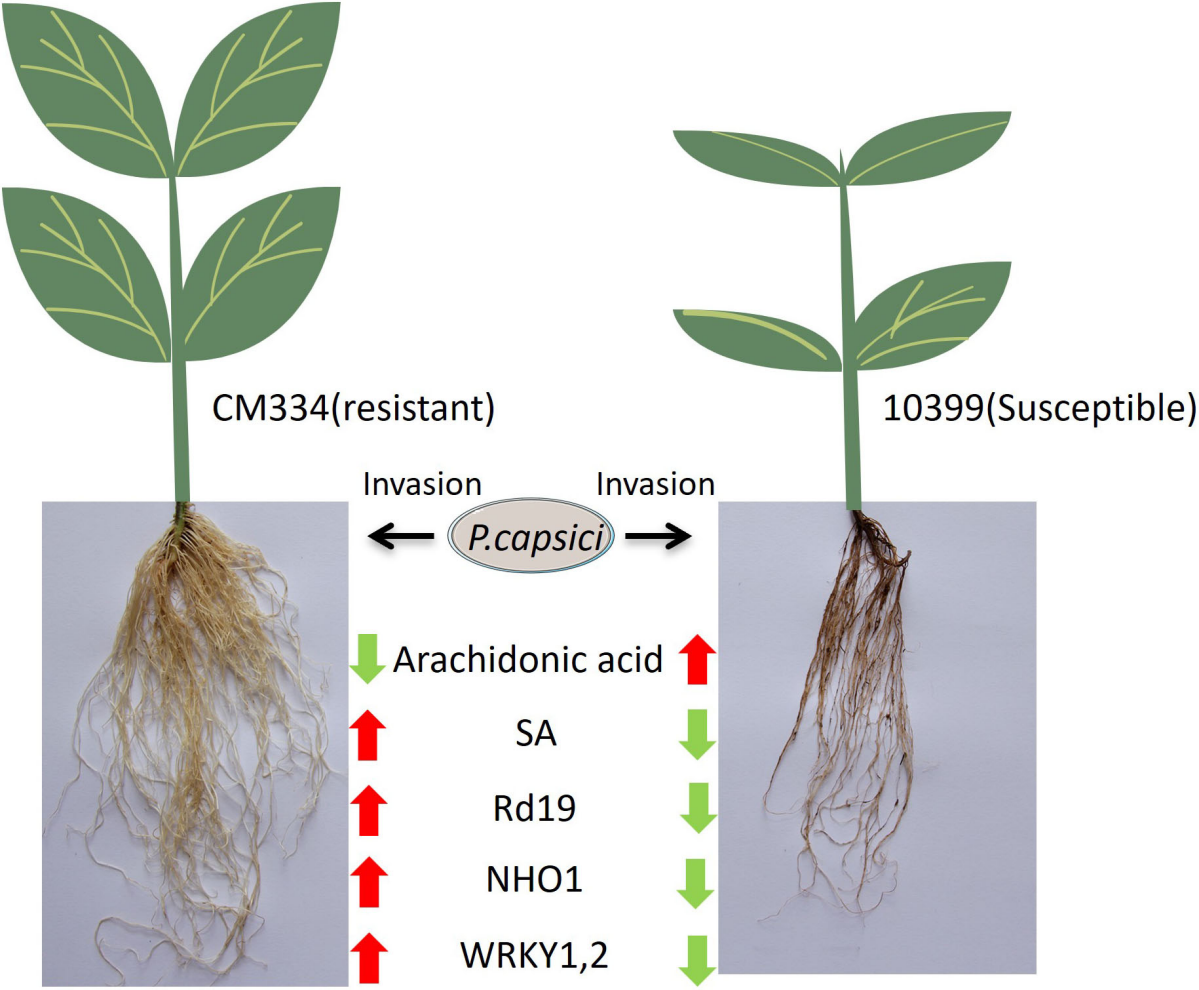
Schematic summarizing the different transcriptome and metabolome profiles in resistant CM334 and susceptible 10399 upon *P. capsici* infection. (A downward green arrow indicates downregulation of a metabolite or protein after infection, while an upward red arrow indicates upregulation).

In summary, arachidonic acid (AA) emerged as the most significant differential metabolite between resistant and susceptible pepper varieties, while the ABC transporter pathway was identified as the primary differential protein pathway. Salicylic acid (SA) exhibited significant upregulation in the resistant variety. Within the plant–pathogen interaction (PPI) pathway, the nonhost resistance protein NHO1, as well as ETI-related proteins Rd19, WRKY1, and WRKY2, were significantly upregulated in the resistant variety (**Figure 7**). These findings suggest that both PAMP-triggered immunity (PTI) and effector-triggered immunity (ETI) are activated in the resistant variety but not in the susceptible one. The levels of AA appear to have a substantial impact on resistance, and Rd19, along with WRKY1 and WRKY2, may represent key proteins in ETI. Future research should focus on validating the resistance functions of Rd19, WRKY1, and WRKY2 in peppers.

This study, through integrated metabolomics and proteomics analyses, characterized the differences between resistant and susceptible pepper varieties. By identifying potential key metabolites and proteins, this work provides novel insights into the mechanisms of blight resistance in peppers and offers a foundation for the development of resistant cultivars.

## Data Availability

The data presented in the study are deposited in the China National Center for Bioinformation (https://www.cncb.ac.cn/) repository, accession number OMIX012227 and OMIX012236.
