# Supplementary figures and images for: Comparative proteomic and metabolomic analyses reveal resistance mechanisms in Chilli pepper roots of resistant and susceptible varieties to Phytophthora capsici infection
Source: Front Plant Sci. 2025 Oct 20;16:1638114. doi: 10.3389/fpls.2025.1638114 (PMC12583989; doi:10.3389/fpls.2025.1638114)

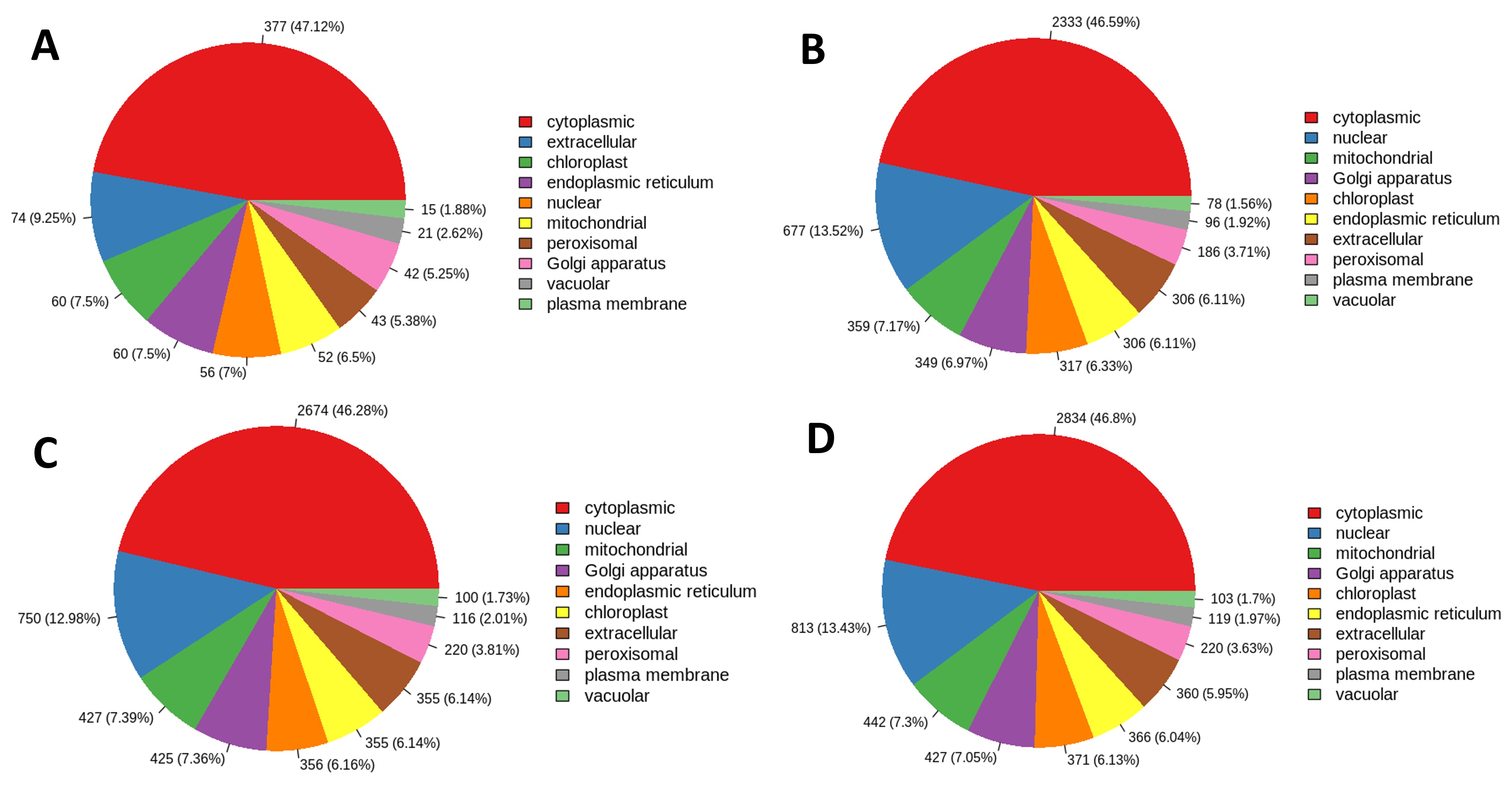

Supplement: Supplementary file 1 [file Image1.jpeg]

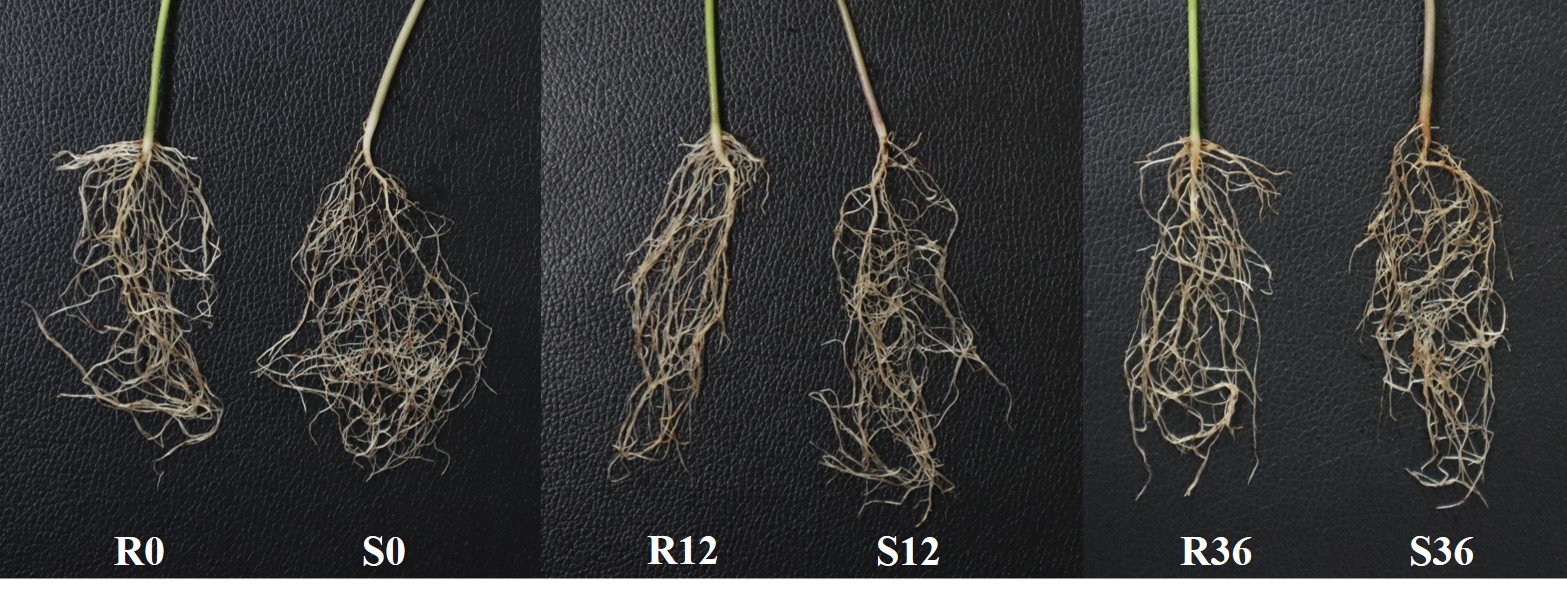

Supplement: Supplementary file 2 [file Image2.jpeg]
