# Supplementary material for: Comparative proteomic and metabolomic analyses reveal resistance mechanisms in Chilli pepper roots of resistant and susceptible varieties to Phytophthora capsici infection
Source: Front Plant Sci. 2025 Oct 20;16:1638114. doi: 10.3389/fpls.2025.1638114 (PMC12583989; doi:10.3389/fpls.2025.1638114)
Supplement: Supplementary file 3 [file Table1.docx]

| Gene name | Protein ID | Gene ID | Antisense Primer | Sense Primer |
| --- | --- | --- | --- | --- |
| Rd19 | tr\|A0A2G3AH41\|A0A2G3AH41_CAPAN | CA12g06050 | GCCGCTTTCTTTGTTCAGTT | CTCTGCGTGGTTGCGTCTT |
| WRKY1 | tr\|A0A1U8HAR7\|A0A1U8HAR7_CAPAN | CA07g21030 | AACTTGTTTCTGCCCGTA | ACTCGCTTCAATCTCACC |
| WRKY2 | tr\|A0A1U8HDR2\|A0A1U8HDR2_CAPAN | CA07g10930 | GACCACCATTACTATCACC | AAATGAGACGACCAGACA |
| NHO1 | tr\|A0A1U8FUZ6\|A0A1U8FUZ6_CAPAN | CA03g32600 | ATGGCATTGTAAAGAGGAAGG | GATCTGTATGGCGAGGGC |
| WRKY33 | tr\|D7NPU8\|D7NPU8_CAPAN | CA03g32070 | GCACCCACAAGAGTATTT | CGATTCTAGTAGCGACAT |
| Actin | AAS78671.1 | AY572427 | GCCGTGCTTTCCTTGTAT | TTCCCGTTCAGCCGTGGT |

Talbe S7 Primers used in qRT-PCR validation

Gene name
